# Intronic RNAscope probes enable precise identification of cardiomyocyte nuclei and cell cycle activity

**DOI:** 10.1038/s42003-025-08012-z

**Published:** 2025-04-07

**Authors:** Zhe Yu, Sen Zhang, Julius Bogomolovas, Ju Chen, Sylvia M. Evans

**Affiliations:** 1 Skaggs School of Pharmacy and Pharmaceutical Sciences, University of California San Diego, La Jolla, CA 92093 USA; 2 Department of Pharmacology & Regenerative Medicine, University of Illinois Chicago, Chicago, IL 60612 USA; 3 Department of Medicine, University of California San Diego, La Jolla, CA 92093 USA; 4 Department of Pharmacology, University of California San Diego, La Jolla, CA 92093 USA

**Keywords:** Fluorescence imaging, Cellular imaging

## Abstract

Cardiac regeneration studies have been plagued by technical challenges in unequivocally identifying cardiomyocyte (CM) nuclei in cardiac sections, crucial for accurate identification of cycling CMs. The use of antibodies to sarcomeric proteins is error-prone, the CM specificity of common nuclear markers is controversial, and utilizing genetically modified mouse models poses risk of inducing unintended cardiac phenotypes. The application of RNAscope intronic probes overcomes the above shortcomings. Intronic probes label intronic RNAs within nuclei and can therefore be utilized as a method for nuclear localization. A *Tnnt2* intronic RNAscope probe highly colocalized with Obscurin-H2B-GFP in adult mouse hearts, demonstrating CM specificity. Studies in embryos demonstrated that the *Tnnt2* intronic RNAscope probe labeled CM nuclei that had undergone DNA replication, and remained closely associated with CM chromatin at all stages of mitosis, even with nuclear envelope breakdown. The efficiency, accuracy, and perdurance of the *Tnnt2* intronic RNAscope probe even with nuclear envelope breakdown facilitated reliable investigation of dynamics of DNA synthesis and potential mitoses in CMs in both border and infarct zones after myocardial infarction (MI). Furthermore, we designed *Myl2* and *Myl4* intronic RNAscope probes, which labeled ventricular and atrial CM nuclei, respectively, and may help identify CM subtypes generated in vitro.

## Abstract (web summary)

Intronic RNA probes precisely identify cardiomyocyte nuclei or chromatin throughout the cell cycle during embryonic cardiomyocyte proliferation and precisely identify cardiomyocyte nuclei in adult heart, enabling cardiac regeneration studies.

## Introduction

Adult cardiomyocytes (CMs) have traditionally been considered to be postmitotic. Embryonic and neonatal hearts can increase CM numbers through CM proliferation, whereas adult hearts increase mass primarily through hypertrophic growth of CMs^1–3^. However, recent studies have suggested that a low level of CM turnover exists in adult mammalian hearts, which can be stimulated to a higher level by heart injuries, such as myocardial infarction (MI)^4–6^. Also, these studies suggested that newly generated CMs originate from pre-existing CMs^5,6^.

A critical challenge in the field of cardiac regeneration is to efficiently and specifically identify CM nuclei in cardiac sections^1,7–12^. The first step to confirm mammalian CM proliferation at baseline or after cardiac injury is to capture CMs which are engaged in cell cycle re-entry, utilizing probes to detect cell cycle specific markers in the nucleus of CMs. Owing to the cellular complexity of cardiac tissue, with a plethora of non-CM nuclei surrounding CMs, attributing any cell cycle specific markers to CM nuclei is challenging. Several different strategies have been used to identify CM nuclei in cardiac sections.

Sarcomeric proteins are specific markers for CMs. In most studies, authors have used antibodies to sarcomeric proteins including α-actinin, myosin heavy chains, and cardiac troponins (C, I, and T) together with DNA dyes such as DAPI to identify CM nuclei in cardiac sections using confocal microscopy. CM nuclei showing colocalization between sarcomeric proteins and cell cycle indicators such as Ki67, phosphorylated histone H3 (pH3), Aurora B, Anillin, or thymidine analogs like 5′-bromo-2′-deoxyuridine (BrdU) or 5-Ethynyl-2′-deoxyuridine (EdU) are considered as cell cycle active^1,7^. However, in adult heart, although CMs occupy 70% of the volume, they only account for 20–30% of the total nuclei. CM nuclei can be juxtaposed to interstitial nuclei, making it difficult to distinguish between CM and interstitial nuclei. Utilizing antibodies to sarcomeric proteins as markers, the sensitivity and specificity of CM nuclei identification has been estimated to be 43% and 89%, respectively. Even combined with wheat germ agglutinin (WGA) staining to outline cell membranes, sensitivity and specificity are only increased to 65% and 97%, respectively^7^.

Another approach is to utilize antibodies to endogenous nuclear markers such as Nkx2.5^13–15^, Gata4^7,13^, Mef2c^16^, and PCM1^8,17–19^ to localize CM nuclei in cardiac sections. However, the application of these markers still has limitations. Gata4 and Mef2c have been shown to be expressed in non-CMs^7,13,16,20^. Nkx2.5 is reasonably specific to adult CMs, although its expression pattern after cardiac injuries is unclear^1,20^. Additionally, expression levels of Nkx2.5 in adult CM nuclei is quite low, making it difficult to detect without an effective antibody. PCM1 is a centrosomal matrix protein that is widely expressed in various cell types, and its specificity to CM nuclei is still controversial^21^. One study suggested that localization of PCM1 to the CM nuclear envelope might be associated with loss of proliferative capacity^19^. Thus, if a small subset of adult CMs retain proliferative potential, in these CMs, PCM1 may be localized to centrosomes. Many of the above markers may fail to be visualized during mitosis upon nuclear envelope breakdown, making it challenging to capture CMs undergoing mitosis. Additionally, post MI, staining for all nuclear antigens in the BZ and IZ is challenging due to high background autofluorescence^8^. However, identification of cell cycle active CMs in these regions is particularly important since the cell cycle activity of CMs in the border zone (BZ) and infarct zone (IZ) is stimulated to a much higher degree compared with the remote zone (RZ)^5,6,22–24^.

In addition to the above approaches, some researchers have established genetically modified mouse models to label CM nuclei, such as the Obscurin-H2B-GFP expressing mouse^25,26^, the αMHC-H2B-mCherry mouse^8,27^, and the MHC-nLAC mouse^22^. However, utilizing these mouse lines requires costs associated with mouse colony maintenance, with other considerations being mouse strain background concerns, or potential cardiac phenotypes consequent to the genetic manipulations.

In eukaryotic cells, immature strands of messenger RNA, also known as pre-mRNAs, are first transcribed from DNA templates in the nucleus. These pre-mRNAs may contain both introns and exons, and must undergo a series of modifications including splicing before being transported out of the nucleus^28^. Thus, labeling of the intronic regions of the pre-mRNAs would be expected to allow for nuclear labeling. A previous study demonstrated that fluorescently labeled antisense probes to HER2 intronic transcripts localized to nuclei^29^. Recently, a novel RNA in situ hybridization (ISH) technique, named RNAscope, utilizing a unique probe design strategy that enables simultaneous signal amplification and background suppression for single-molecule visualization while preserving tissue morphology, has been employed in many studies^30^. RNAscope utilizes probe pairs, referred to as ‘ZZ’ pairs, with each pair targeting approximately 50 bases of the target mRNA^31^. RNAscope also has been used in the field of cardiac research^32,33^. Therefore, we hypothesized that designing intronic RNAscope probes for intronic RNA encoding sarcomeric proteins would specifically identify cardiac nuclei.

We have previously applied intronic RNAscope probes for identification of CM nuclei in adult heart sections at baseline and post MI^24^, where we found no evidence to support substantial CM proliferation. In this study, to further investigate the behavior of the *Tnnt2* intronic RNAscope probe in CMs undergoing proliferation, we utilized heart tissue from wildtype embryos or genetically labeled Fucci2a cell cycle indicator embryos^34^ at E9.5, when CMs are proliferating. Results demonstrated that the *Tnnt2* intronic RNAscope probe can specifically detect CM nuclei undergoing DNA synthesis, and CM nuclei or chromatin at each stage during mitosis, including after nuclear envelope breakdown. These results demonstrated that the intronic probe can be utilized with confidence to detect any CMs actively engaged in mitosis. Capitalizing on the efficiency, accuracy, and ability of the *Tnnt2* intronic RNAscope probe to detect CMs in all stages of mitoses, we investigated the time course of DNA synthesis rates and any mitotic activity in CMs post MI in BZ and IZ of adult heart. Finally, we designed and utilized *Myl2* and *Myl4* intronic RNAscope probes to selectively identify ventricular and atrial CM nuclei, respectively. The latter approach should be of great utility to readily identify specific CM cell types generated in vitro from ESCs or iPSCs and to accurately examine their biological activities of interest, including proliferation.

## Methods

### Animal models

Animal studies were conducted in strict compliance with protocol (S04150) approved by the Institutional Animal Care and Use Committee of the University of California, San Diego (UCSD) and the Guide for the Care and Use of Laboratory Animals published by the National Institutes of Health. We have complied with all relevant ethical regulations for animal use. Mice were kept in IVC disposable cages (Innovive), under a 12-h light cycle. We used the FVB/NJ (#001800, The Jackson Laboratory), Obscurin-H2B-GFP^25,26^, Fucci2a^34^, and *XMLC2-Cre*^35^ mouse lines for our study, which have been previously described. All mice used in this study were backcrossed with FVB/NJ mice for a minimum of six generations. For genotyping of transgenic animals, tail biopsies were collected at weaning (3-week-old).

Wild-type or GFP alleles were detected by PCR using the following primers:

OB-F: 5’-CACATCAAGGTAACTGAAGATCC-3’;

OB-R: 5’-CTTGCCAACAGATACCACAAAG-3’;

GFP-R: 5’-CACGAACTCCAGCAGGACCAT-3’.

Fucci2a alleles were detected by PCR using the following primers:

Rosa26-F: 5’-CTCTGCTGCCTCCTGGCTTCT-3’;

Rosa26-R: 5’-CGAGGCGGATCACAAGCAATA-3’;

mT/mG-R: 5’-TCAATGGGCGGGGGTCGTT-3’.

*XMLC2-Cre* were detected by PCR using the following primers:

*XMLC2*- F: 5’-TAGGATGCTGAGAATCAAAATGT-3’;

*XMLC2*- R: 5’-TCCCTGAACATGTCCATCAGGTTC-3’.

### Histology

E9.5 embryos or adult hearts were dissected in RNase-free 1X PBS, fixed in 4% PFA for one hour or overnight at 4 °C, saturated in 5%, 10%, 15%, and 20% sucrose in PBS, and frozen in O.C.T. compound (Tissue-Tek, 4583). Cryosections were prepared using a Leica CM3050 S Cryostat (Leica Microsystems). The section thickness was 8 µm for E9.5 embryos and 16 µm for adult hearts.

For analysis of CM DNA synthesis following MI or Sham surgery, hearts were sectioned at a thickness of 16 µm. Sectioning began at the ligation site for MI hearts or the suture penetration site for Sham hearts. Five sections per heart, each spaced 500 µm apart from the starting site, were selected for analysis. The slides were stained with Sirius Red/Fast Green (Chondrex, 9046) to visualize fibrotic areas. Images were captured using the NanoZoomer Slide Scanner (Hamamatsu Photonics).

### Adult CM isolation

Adult CM isolation was performed based on previously established protocols with modifications^36^. The heart was removed following ketamine anesthesia and rinsed in Krebs-Henseleit buffer B (KHB-B) (118 mM NaCl, 4.8 mM KCl, 25 mM HEPES, 1.25 mM K_2_HPO_4_, 1.25 mM MgSO_4_, 11 mM glucose, pH 7.2). The heart was cannulated through the aorta and perfused on a Langendorff apparatus with KHB solution (5 minutes, 37 °C), then incubated with KHB enzyme solution (0.5 mg/mL Collagenase Type 2, Worthington LS004177; 0.5 mg/mL Collagenase Type 4, Worthington LS004189; 0.04 mg/mL Protease Type XIV, Sigma-Aldrich P5147) for 20 minutes at 37 °C. After digestion, hearts were minced in KHB solution with 2% BSA, gently agitated, then filtered through a 100 μm polyethylene mesh. After settling, CMs were fixed in 4% PFA for 30 min, followed by dehydration with 5-min washes in 50%, 70%, and two consecutive washes in 100% methanol. CMs can be stored in 100% methanol at −20 °C for further use.

### RNAscope fluorescent in situ hybridization

RNAscope fluorescent in situ hybridizations (ISH) were conducted using the RNAscope™ Multiplex Fluorescent Reagent Kit v2 (Advanced Cell Diagnostics, 323100) with several adaptations.

Cryosections were refixed in 4% PFA for 15 min, followed by dehydration with 5-min washes in 50%, 70%, and two consecutive washes in 100% ethanol. Next, sections were treated with hydrogen peroxide for 10 min to inhibit endogenous peroxidase activity and reduce non-specific background staining. Protease III treatment at room temperature for 20 min was used to permeabilize the tissue, allowing probes to penetrate and access target RNA molecules. Probe hybridization was carried out at 40 °C for two hours, followed by sequential hybridizations with the preamplifier, amplifier, and label probes to yield up to 8000 labels per target RNA molecule.

Isolated CMs were rehydrated with 5-minute washes in 70% and 50% methanol in PBT (PBS containing 0.1% Tween 20), followed by two consecutive washes in PBT. Protease III treatment was performed at 40 °C for 15 min to permeabilize CMs. Probe hybridization was performed overnight at 40 °C, followed by sequential hybridizations with the preamplifier and amplifier, similar to the procedure used for cryosections.

For EdU analysis, after RNAscope ISH cryosections of heart tissue were stained with the Click-it EdU kit (ThermoFisher, C10339) according to manufacturer’s protocol.

For samples co-stained with antibodies after RNAscope ISH, sections were permeabilized in PBS-0.1% Triton for 10 min and incubated for one hour at room temperature in blocking solution (PBS-0.1% Triton, 10% donkey serum, 5% BSA) before overnight primary antibody incubation at 4 °C. Sections were washed three times in PBS-0.1% Triton and incubated with AlexaFluor-conjugated secondary antibodies (Thermo Fisher Scientific) diluted 1:300 in blocking solution, with DAPI (1:1000, Life Technologies) used for nuclear labeling.

Catalog numbers for RNA-scope probes (ACD bio) used in this study: RNAscope™ Probe-Mm-Tnnt2-O1, Cat No. 880391, 20 ZZ pairs targeting region 807-1739 of NC_000067.6:135836315-135852268; RNAscope™ Probe-Mm-Myl2-intron6-C2, Cat No. 1056851-C2, 15 ZZ pairs targeting region 2-1365 of NC_000071.7:122243267-122244646; RNAscope™ Probe-Mm-Myl4-intron3-C2, Cat No. 1056861-C2, 20 ZZ pairs targeting region 275-1559 of NC_000077.7:104468704-104470923; RNAscope™ Probe- Mm-Tnnt2-C2, Cat No. 418681-C2, 18 ZZ pairs targeting region 184-1173 of NM_011619.3.

Catalog numbers for primary antibodies: Anti-GFP antibody (Abcam, ab13970); Anti-Phospho-Histone H3 (Ser10) antibody (MilliporeSigma, 06-570).

Catalog numbers for secondary antibody: Donkey anti-Rabbit IgG (H + L) Highly Cross-Adsorbed Secondary Antibody, Alexa Fluor™ Plus 488 (Thermo Fisher Scientific, A32790).

The detailed step-by-step protocol is available at Protocols.io: 10.17504/protocols.io.eq2ly6mbmgx9/v1

### Imaging and analysis using confocal microscopy

Image acquisition was performed using a Leica SP8 Confocal with Lightning Deconvolution. The IZ was confirmed as the *Tnnt2*-negative region, and the BZ was identified by selecting areas where the infarct scar was at the edge of the image, limiting quantification to CMs within the BZ. Image quantification was performed using ImageJ, and editing was completed with Adobe Illustrator.

### Permanent ligation of the left anterior descending artery (LAD)

8-week-old FVB/NJ male mice were anesthetized with a cocktail of ketamine (50 mg/kg) and xylazine (5 mg/kg) intraperitoneal (IP) injection for initial induction and then isoflurane (0.75–1.5%) for complete induction and maintenance of anesthesia for the entire surgical procedure. Anesthesia was monitored by visual examination of spontaneous movement, blinking, breathing level and frequency, and the response to a gentle stimulus, i.e. toe/foot squeeze. Care was taken to ensure the mouse was maintained at a deep surgical condition of anesthesia to avoid the risk of discomfort, distress, or pain for entire procedure. Mice were intubated with a pressure ventilator throughout the surgical procedure with isoflurane. A skin incision was made from the midsternal line toward the left armpit, and the chest opened with a 1 cm lateral cut along the left side of the sternum, cutting between the 3rd and 4th ribs to expose the left ventricle of the heart. The ascending aorta and main pulmonary artery were identified; the left anterior descending (LAD) was located as it traverses the anterior wall of the heart, between the left and right ventricles. LAD occlusion was performed by tying a 7-0 or 8-0 prolene suture. Blanching of the territory of perfusion of the LAD, along with acute ST segment elevation on limb-lead electrocardiographic leads, were observed in order to certify the occlusion of the vessel. Once the mouse was hemodynamically and rhythmically stable, the ends of the suture were ligated, and the chest was closed with one layer of suture of 6-0 braided black silk through the chest wall and the skin was closed with 6-0 suture. Air was evacuated by increasing the ventilation rate 20–30% more for 5 minutes to prevent Pneumothorax. Sham operated mice underwent the same procedure without ligation of the LAD. Post-operative recovery was usually uneventful, and the animals were observed closely until they were stable and conscious. Buprenorphine (0.1 mg/kg) was given right after anesthesia induction. Additional Buprenorphine was given every 4–8 h for up to 48 h if there was any sign of the pain and distress.

### EdU administration

Mice were IP injected with 200 μg of EdU (in sterile PBS) once per day for four consecutive days. The first day of EdU injections started on days 2, 4, 6, 8, 10, 12, 14, 16, and 30 post MI surgeries. Animals were euthanized 2 days after the final EdU injection.

### Statistics and reproducibility

Replicates and statistical tests are described in the figure legends. No statistical methods were used to predetermine sample size. For LAD ligation, animals were randomly assigned to either the Sham or MI group. The order of Sham or MI surgeries was randomized, and all animals were housed in the same room and on the same rack to minimize environmental confounders. During the allocation, the researchers performing the surgeries were unaware of the assigned timepoints for the animals. Animals were included only if MI was successfully induced during surgery, as evidenced by infarction. Animals without infarction were excluded from the study. This criterion was established a priori. Three mice in the MI groups were excluded due to almost no infarction being generated, indicating surgery failure. In addition, the images were analyzed with the timepoints blinded by randomizing the order of the samples. Outcome assessors were not aware of the experimental groups until the data were analyzed and results were generated. All experimental results were analyzed with at least three independent embryos or adult hearts, unless otherwise specified. Data are presented as mean ± SD or mean ± SEM, as indicated in the figure legend. *P* < 0.05 was considered to be statistically significant. Negative binomial regression was performed by SPSS 28. One-way ANOVA was performed by GraphPad Prism 10.1.2. *P < 0.05. **P < 0.01. ***P < 0.001. ****P < 0.0001.

### Reporting summary

Further information on research design is available in the Nature Portfolio Reporting Summary linked to this article.

## Results

### A *Tnnt2* intronic RNAscope probe labels adult CM nuclei efficiently and specifically

**Fig. 1:**
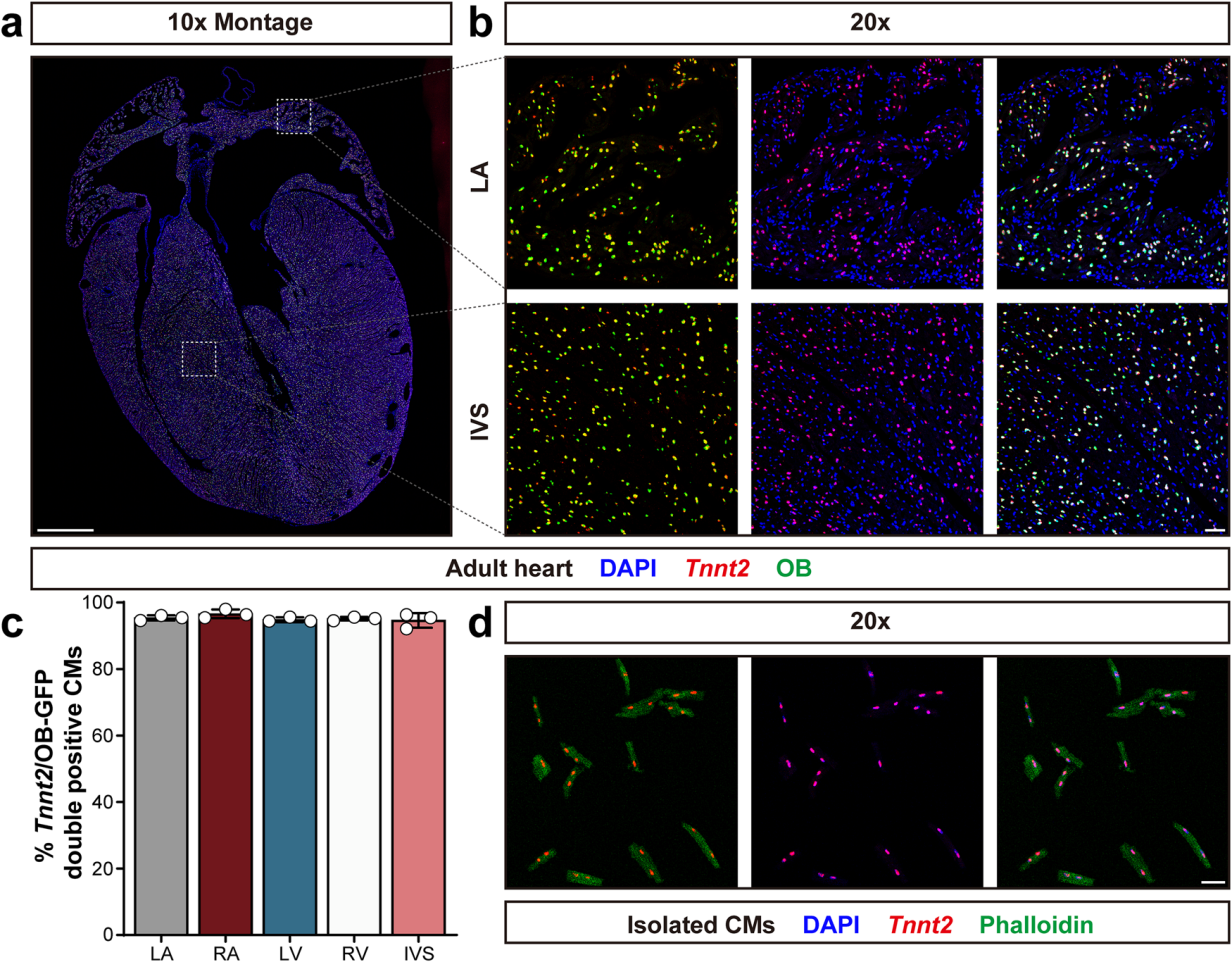
*Tnnt2* intronic RNAscope probe labels CM nuclei efficiently and specifically. **a** Both *Tnnt2* intronic RNA and Obscurin-H2B-GFP fluorescent signals were detected throughout the hearts. Scale bar, 1 mm. **b** Representative figures demonstrated *Tnnt2* intronic RNAs showed a high degree of colocalization with Obscurin-H2B-GFP. Scale bar, 100 μm. **c** More than 95% of labeled CM nuclei are positive for both *Tnnt2* intronic RNAscope probe and Obscurin-H2B-GFP. Three hearts were used for analysis, and in each heart three photos were taken for each region: LA, RA, LV, RV, and IVS. Total count of CM nuclei: 1443 in LA, 1264 in RA, 1451 in LV, 1901 in RV, and 1826 in IVS. Negative binomial regression was performed to determine statistical significance. Data are presented as mean ± SD. **d** *Tnnt2* intronic RNAscope probe stained isolated CMs efficiently. CMs were isolated using the Langendorff method from three 8-week-old female FVB/NJ mice. In each mouse, 500 CMs were observed, and all were successfully labeled with the *Tnnt2* intronic RNAscope probe. Scale bar, 100 μm. LA left atrium, RA right atrium, LV left ventricle, RV right ventricle, IVS interventricular septum.

The Obscurin-H2B-GFP expressing mouse line has been used to localize CM nuclei in previous studies^25,26^. Thus, initially we used *Obscn*^GFP/GFP^ reporter mice to test the efficiency and accuracy of the *Tnnt2* intronic RNAscope probe in CM nuclei. Hearts from 8-week-old *Obscn*^GFP/GFP^ male mice were harvested and cryo-sectioned. Then RNAscope fluorescence assays with the *Tnnt2* intronic RNAscope probe, combined with immunofluorescent antibody staining for GFP were performed for cardiac sections. Strong signals for *Tnnt2* intronic RNA and Obscurin-H2B-GFP were observed throughout the heart (Fig. 1a). Also, the *Tnnt2* intronic signal showed a high degree of colocalization with Obscurin-H2B-GFP (Fig. 1b). The frequency of CM nuclei double-labeled with *Tnnt2* intronic RNAscope probe and GFP fluorescent signal was calculated using the total number of CM nuclei labeled with either *Tnnt2* intronic RNAscope probe or GFP as the reference. Results showed that more than 95% of total CM nuclei were double-labeled with *Tnnt2* intronic RNAscope probe and GFP fluorescent signals. No significant difference in frequency was observed between the different regions of the heart (Fig. 1c). In contrast, when using the normal exonic *Tnnt2* RNAscope probe, *Tnnt2* exonic RNA was detected throughout the cytoplasm, interspersed with numerous interstitial nuclei, making it difficult to distinguish between CM and interstitial nuclei (Supplementary Fig. 1). The *Tnnt2* intronic RNAscope probe was also able to stain isolated CMs efficiently (Fig. 1d). Thus, the *Tnnt2* intronic RNAscope probe labeled CM nuclei efficiently and accurately.

### In embryonic hearts, *Tnnt2* intronic RNAscope probe identifies CM nuclei, even during mitosis

**Fig. 2:**
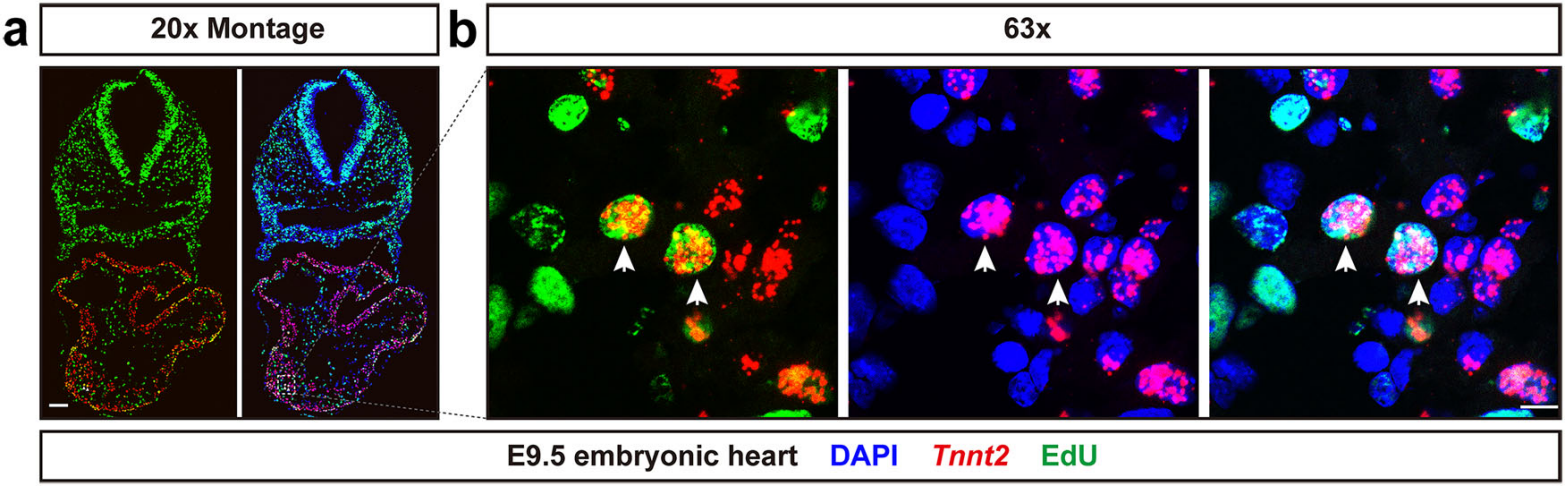
*Tnnt2* intronic RNAscope probe labels CM nuclei that have undergone DNA synthesis. Pregnant FVB/NJ mice were injected with 30 mg/kg of EdU at E9.5. Embryos were harvested one hour later. **a** *Tnnt2* intronic RNA and EdU fluorescent signals were detected throughout the heart tube at E9.5. Scale bar, 100 μm. **b** Representative images showed colocalization of fluorescent signals from *Tnnt2* intronic RNAs and EdU in CM nuclei. Scale bar, 10 μm.

Adult mammalian CMs are terminally differentiated and are among the least regenerative cell types. However, whether proliferation of CMs occurs in pathological conditions such as ischemic injuries caused by MI, is still controversial^4–6^. We aim to use this *Tnnt2* intronic RNAscope probe to address these questions. Since embryonic CMs can actively proliferate, we first tested whether this probe can be used to detect embryonic CM nuclei undergoing DNA synthesis during S phase prior to cell division. Pregnant FVB/NJ female mice received IP injections of 30 mg/kg of EdU at E9.5. One hour later, embryos were isolated, fixed with 4% PFA, and cryo-sectioned. RNAscope fluorescent assays with the *Tnnt2* intronic RNAscope probe, combined with staining for EdU were performed. Robust *Tnnt2* intronic probe and EdU fluorescent signals were detected in the developing S-shaped heart tube (Fig. 2a). Further analyses showed colocalization of *Tnnt2* intronic RNA and EdU fluorescent signals in CM nuclei in the forming heart tube (Fig. 2b). Thus, the *Tnnt2* intronic RNAscope probe is capable of labeling embryonic CM nuclei which have undergone DNA synthesis.

**Fig. 3:**
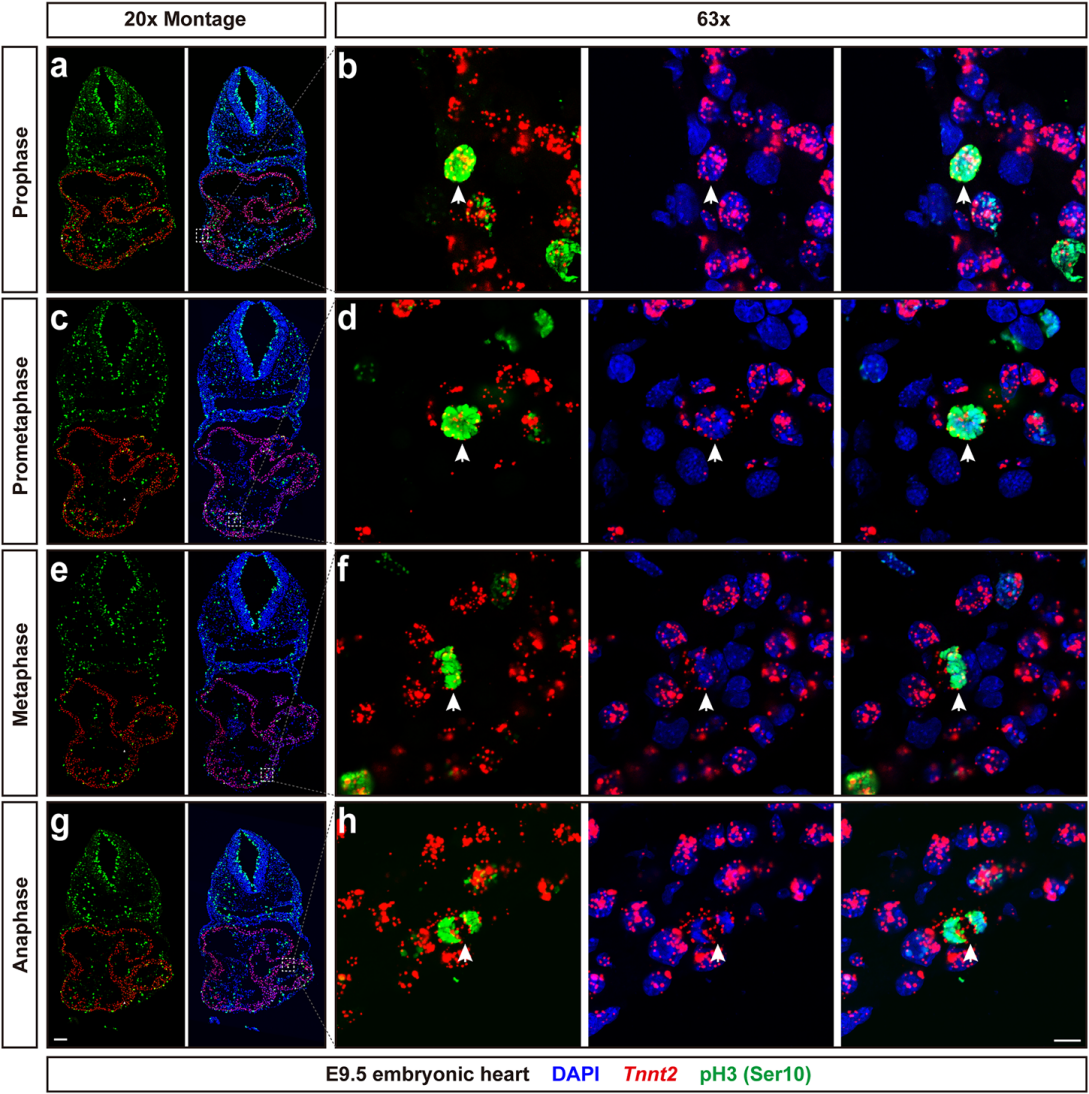
*Tnnt2* intronic RNAscope probe identifies CM nuclei or chromatin at all stages of mitosis. CM nuclei were labeled with the *Tnnt2* intronic RNAscope probe, while chromosome structures were marked with pH3 (Ser10). DNA in nuclei was stained with DAPI. Representative CM nuclei at each stage of mitosis are shown by arrows. **a**, **b** Representative image of prophase CM nucleus. pH3 fluorescent signals were spread throughout the nucleus. Fluorescent signals from *Tnnt2* intronic RNAs colocalized with fluorescent signals from pH3. **c, d** Representative image of prometaphase CM nuclei. At this stage, condensed chromosomes were clearly labeled by pH3. Fluorescent signals from *Tnnt2* intronic RNAs and pH3 remained colocalized. **e**, **f** Representative pictures of metaphase CM nuclei. The condensed chromosomes in the metaphase plate, labeled with pH3, were clearly visible. *Tnnt2* intronic RNAs overlapped with the chromosomes, and were also distributed around them. **g**, **h** Representative image of anaphase CM nuclei. The two sets of chromosomes (sister chromatids) were labeled clearly by pH3 during anaphase. Similar to metaphase, colocalization between *Tnnt2* intronic RNAs and pH3 fluorescent signals was detected, along with distribution of *Tnnt2* intronic RNAs surrounding the chromosomes. Scale bar for 20x Montage, 100 μm; Scale bar for 63x CM nuclei, 10 μm.

Our previous results showed that in quiescent adult CMs, *Tnnt2* intronic RNAs were colocalized with CM nuclei. Here, we tested whether the *Tnnt2* intronic RNAscope probe could label embryonic CMs undergoing mitosis. We were particularly interested to investigate expression of the *Tnnt2* intronic RNA in metaphase and anaphase, when breakdown of the nuclear envelope has occurred. RNAscope fluorescence assays with the *Tnnt2* intronic RNAscope probe combined with fluorescent immunostaining for phosphorylated histone H3 (pH3, Ser10) were performed. The nuclei, or condensed chromosomes, were also labeled by DAPI. pH3 is a protein expressed in the nuclei of cells during M-phase, or mitosis, of the cell cycle. Expression of pH3 has been used to monitor the progression of mitosis. Scattered foci of pH3 are observed in G2 phase^37^. As chromatin condenses, pH3 begins to accumulate^37,38^. Strong expression of pH3 was observed in embryonic CM nuclei at prophase (Fig. 3a, b). Nuclear envelope breakdown begins to occur at late prophase. Chromosomes become visible at prometaphase, and pH3 staining along the chromosomes was observed (Fig. 3c, d). Colocalization between *Tnnt2* intronic RNA, pH3, and chromosomes (DAPI) was maintained at both prophase and prometaphase. At metaphase, condensed chromosomes aligned at the metaphase plate (Fig. 3e, f). At anaphase, chromosomes begin to separate towards opposite poles (Fig. 3g, h). At both metaphase and anaphase, colocalization between pH3 and chromosomes (DAPI) could be observed. *Tnnt2* intronic RNAs were observed overlapping with chromosomes, with additional diffuse staining surrounding the chromosomes (Fig. 3e–h). Thus, even with nuclear envelope breakdown, the *Tnnt2* intronic probe remained associated with CM chromatin, thus allowing for CM identification throughout mitosis.

**Fig. 4:**
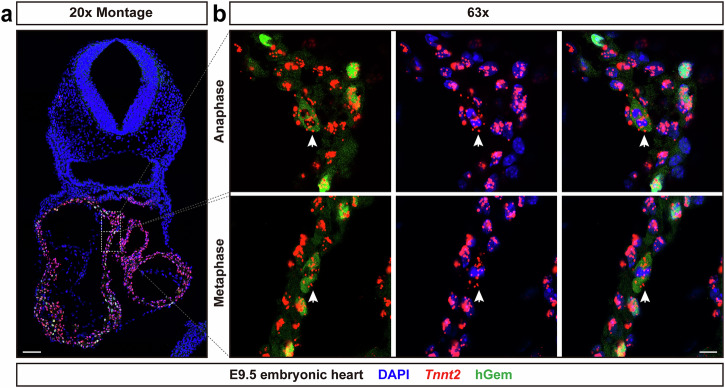
Fucci2a mouse model further demonstrates that the *Tnnt2* intronic RNAscope probe labels CM chromatin during mitosis. **a** Expression of *Tnnt2* intronic RNAs and mVenus-hGem was observed throughout the E9.5 heart tube. Scale bar, 100 μm. **b** mVenus-hGem fusion protein displayed peri-chromosomal localization after nuclear envelope breakdown at metaphase and anaphase. Representative images showed colocalization between *Tnnt2* intronic RNAs and DAPI-labeled CM chromosomes at both metaphase and anaphase. *Tnnt2* intronic RNAs were also distributed around the CM chromosomes, as indicated by overlap with the mVenus-hGem fluorescent signals. Scale bar, 10 μm.

A fluorescent ubiquitination-based cell cycle indicator (Fucci) mouse has been developed that utilizes two antiphase oscillating proteins to visualize cell cycle progression^39^. To further confirm the expression pattern of *Tnnt2* intronic RNAs at both metaphase and anaphase, we crossed *XMLC2-Cre*^35^ male mice with Fucci2a^34^ female mice, and harvested E9.5 embryos to monitor cell cycle progression in CMs. Fucci2a uses red and green fluorescent proteins fused to hCdt1 and hGeminin, respectively^34^. When activated by *XMLC2-Cre*, CMs appear red during G1, yellow during early S, and green during late S and G2. The green fluorescence decreases dramatically during mitosis^40–42^. However, the mVenus-hGem fusion protein displays peri-chromosomal localization after nuclear envelope breakdown at metaphase and anaphase^41,43^, allowing us to distinguish CMs in mitosis. We identified CMs at metaphase and anaphase (Fig. 4a). Similar with previous results (Fig. 2), *Tnnt2* intronic RNAs partially overlapped with DAPI stained CM chromatin, as well as localizing peri-chromosomally. *Tnnt2* intronic RNA probes in fact overlapped with the mVenus-hGem fusion protein at these stages (Fig. 4b, Supplementary Fig. 2). Altogether, our results demonstrated that the *Tnnt2* intronic RNAscope probe identifies CM nuclei/chromatin during each phase of mitosis.

### In adult heart, *Tnnt2* intronic RNAscope probe reveals dynamics of CM EdU incorporation post MI, with no evidence of CM progression through mitosis

**Fig. 5:**
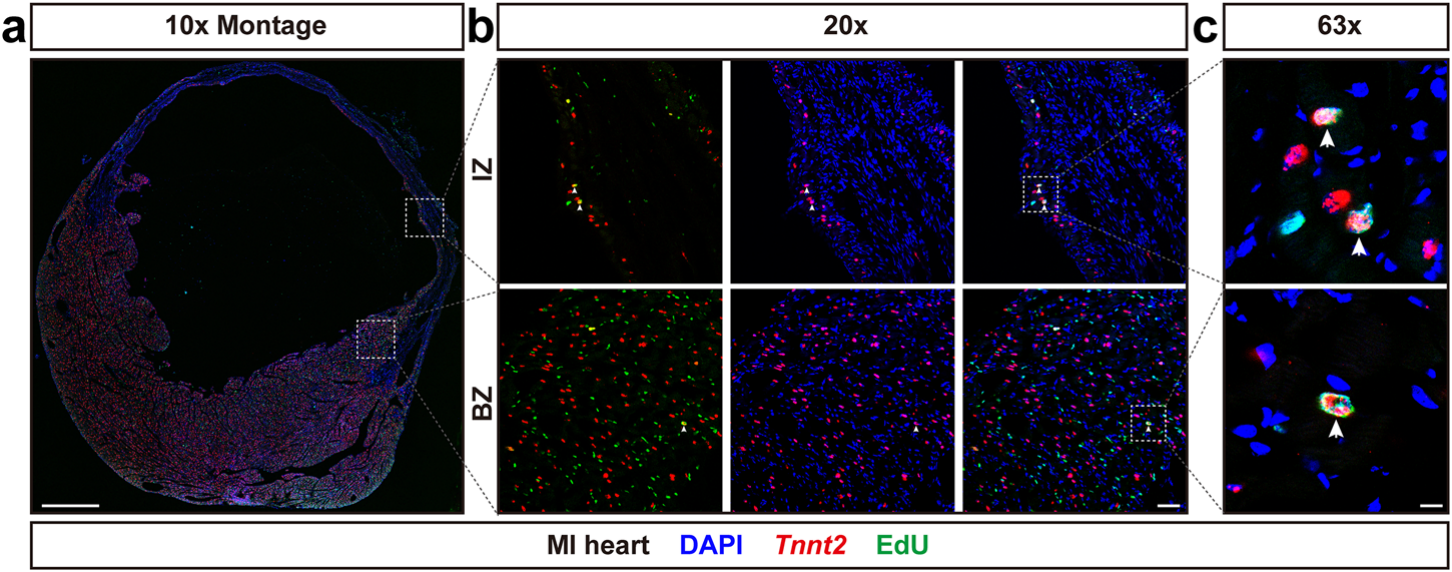
*Tnnt2* intronic RNAscope probe labels CM nuclei that have undergone DNA synthesis. Mice received 200 μg EdU once per day between days 10 to 13 post MI surgeries. Hearts were harvested two days later for histological analyses. **a** Overview of the BZ and IZ in cardiac section. Scale bar, 1 mm. **b** Colocalization of *Tnnt2* intronic RNA and EdU fluorescent signals was observed in both BZ and IZ. Scale bar, 100 μm. **c** Enlarged view of the colocalization between *Tnnt2* intronic RNA and EdU fluorescent signals. Scale bar, 10 μm. BZ border zone, IZ infarct zone.

Previous studies have revealed that post MI, a small percentage of CMs in BZ and IZ may undergo DNA synthesis in response to ischemic injury^5,6,44^. To capitalize on the utility of our *Tnnt2* intronic probe to efficiently capture these rare CMs, we utilized this approach to more fully investigate CM DNA synthesis post MI. Left anterior descending artery (LAD) ligation was performed for 8-week-old FVB/NJ male mice. Mice were IP injected with 200 μg EdU once a day between days 10 and 13 post MI, and hearts were harvested, fixed, and cryo-sectioned for histological analyses on day 15 post MI. RNAscope fluorescent assay with *Tnnt2* intronic probe combined with EdU staining was performed. CM nuclei with *Tnnt2* intronic RNA and EdU fluorescent signals were observed in both BZ and IZ (Fig. 5a, b).

**Fig. 6:**
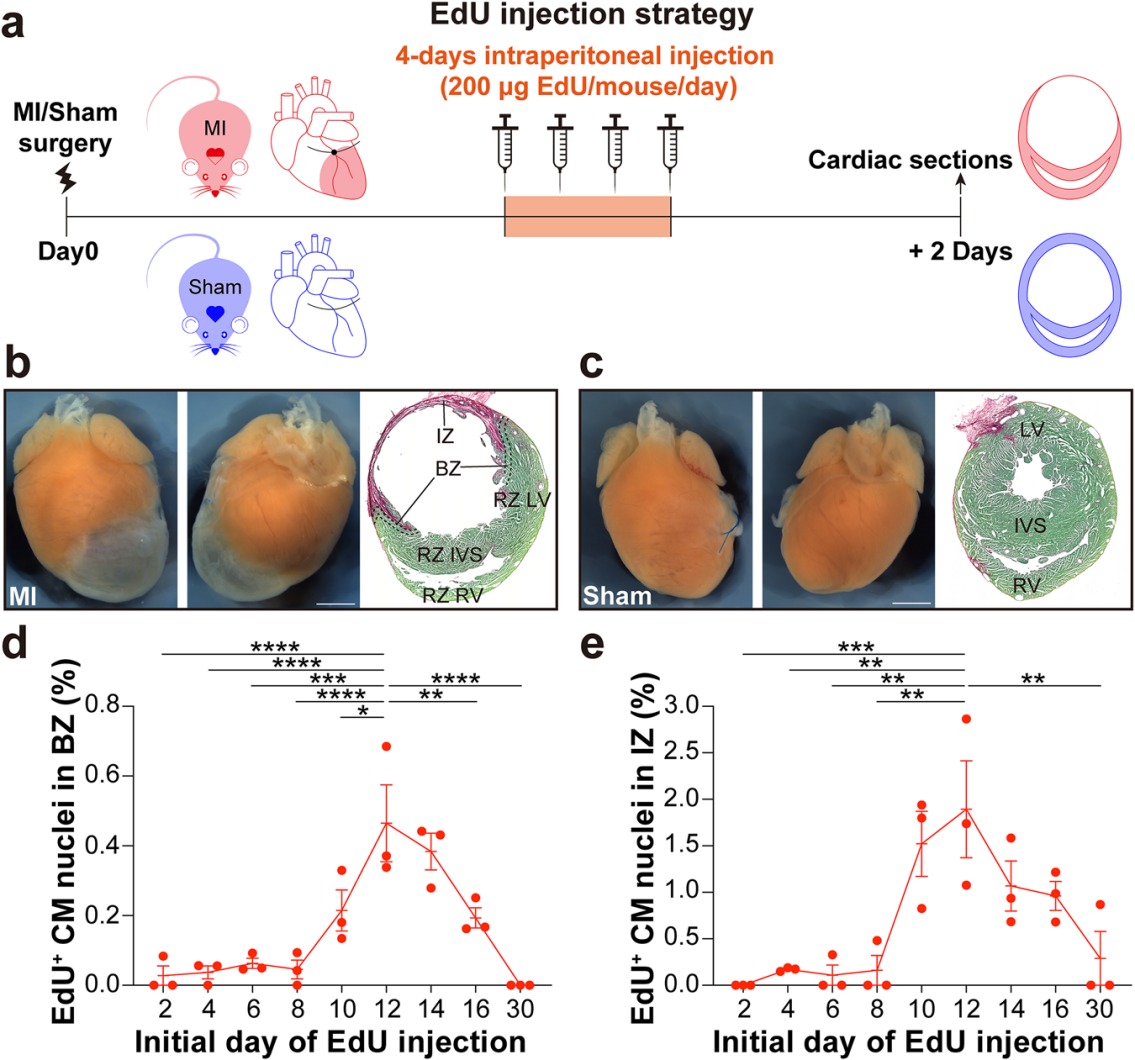
Time Course of EdU incorporation in BZ/IZ CM nuclei post MI surgeries. **a** Schematic of EdU injection strategy. Mice were subjected to MI or Sham surgery, and were then injected once per day with 200 μg EdU for four consecutive days post MI. Initial EdU injections were performed on day 2, 4, 6, 8, 10, 12, 14, 16, or 30 post MI. Hearts were harvested two days following final EdU injection, and cryo-sectioned for histological analyses. **b** Representative images of an MI heart and corresponding sections. The IZ is labeled with Sirius Red. The locations of the BZ, IZ, RZ LV, RZ IVS, and RZ RV are indicated in the figure. **c** Representative images of a Sham heart and corresponding section. The locations of the LV, IVS, and RV are indicated in the figure. Scale bar, 1 mm. **d** Dynamics of BZ CM nuclei EdU incorporation post MI (*n* = 3 for each timepoint). **e** Dynamics of IZ CM nuclei EdU incorporation post MI (*n* = 3 for each timepoint). *P < 0.05; **P < 0.01; ***P < 0.001; ****P < 0.001. One-way ANOVA was performed for P value calculation, followed by Bonferroni correction for multiple comparisons. Data are presented as mean ± SEM. BZ border zone, IZ infarct zone, RZ remote zone.

BrdU and EdU have been used to label DNA synthesis in CM nuclei in a series of studies. However, the timing and duration of BrdU and EdU administration vary across studies^44–47^, and the dynamics of DNA synthesis in CM nuclei post MI has not been comprehensively investigated. Thus, we conducted a time-course study involving a 4-day EdU injection regimen (Fig. 6a). Initially, we performed LAD ligation for 8-week-old FVB/NJ male mice. Sham-operated mice underwent the same procedure as the surgical group, but without LAD ligation. Mice were IP injected with 200 μg of EdU once per day for four consecutive days. We conducted experiments at nine different timepoints, with EdU injections starting on days 2, 4, 6, 8, 10, 12, 14, 16, and 30 post MI surgery. Hearts were harvested two days after the final EdU injection to allow sufficient time for any potential CM proliferation to complete. Then hearts were fixed and cryo-sectioned (Fig. 6b, c). RNAscope fluorescent assays with the *Tnnt2* intronic probe combined with EdU staining were performed for both Sham and MI hearts.

Colocalization of *Tnnt2* intronic RNA and EdU fluorescent signals in CM nuclei were examined in left ventricle (LV), right ventricle (RV), and interventricular septum (IVS) in Sham hearts, as well as in BZ, IZ and RZ (including the residual LV, RV, and IVS) in MI hearts. EdU incorporation in CM nuclei was observed only in MI hearts (Supplementary Table 1) and rarely in Sham hearts (Supplementary Table 2). Intriguingly, although the frequency of EdU^+^ CM nuclei in the IZ was higher than that in the BZ, the dynamics of EdU incorporation in both regions was similar (Fig. 6d, e). Very low frequency of EdU incorporation in CM nuclei was observed for regimens beginning injections at day 2 through day 8 (the latter harvested after final injection at day 11; less than 0.06% in BZ, and less than 0.17% in IZ). Maximal EdU incorporation occurred during days 12-15 post MI (0.46% in BZ and 1.89% in IZ). EdU incorporation in CM nuclei had significantly decreased by days 16–19 post MI (0.19% in BZ and 0.96% in IZ). One month post MI, a low frequency of EdU^+^ CM nuclei were only observed in the IZ of one heart, but not in the BZ within all three hearts. Thus, most EdU incorporation occurred around day 12-15 in BZ and IZ post MI. EdU incorporation in CM nuclei was observed only rarely in the RZ of MI hearts (Supplementary Fig. 3).

**Fig. 7:**
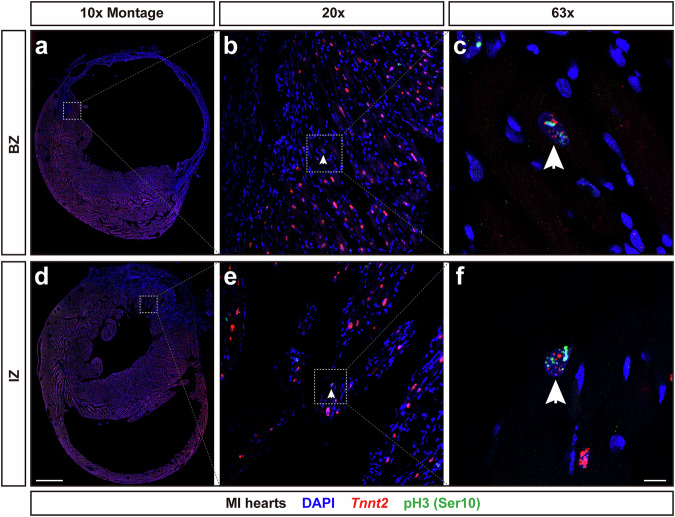
*Tnnt2* intronic RNAscope probe labels CM nuclei expressing pH3. Hearts were harvested at day 13 post LAD surgeries. **a**, **d** Overview of the *Tnnt2* intronic RNA and pH3 fluorescent signals in the BZ and IZ. Scale bar, 1 mm. **b**, **e** Colocalization of *Tnnt2* intronic RNA and pH3 fluorescent signals was observed rarely in either BZ or IZ. Scale bar, 100 μm. **c**, **f** Enlarged view of the colocalization of *Tnnt2* intronic RNA and pH3 fluorescent signals. No condensed chromosomes were observed, this expression pattern of pH3 demonstrated these CMs are in late G2 or early M phase, prior to prometaphase. Scale bar, 10 μm.

We further capitalized on the properties of the *Tnnt2* intronic probe to label CM nuclei undergoing mitosis, and examined whether CM mitosis occurred post MI. We performed LAD ligation again for 8-week-old FVB/NJ male mice. Hearts were harvested at day 13 post MI and RNAscope fluorescence assays with the *Tnnt2* intronic probe combined with fluorescence immunostaining for pH3 were performed. We rarely saw colocalization between *Tnnt2* intronic RNAs and pH3 fluorescent signals (Fig. 7a–f). Only a single pH3 positive CM nucleus was detected in the BZ (0.0076%), or IZ (0.035%) in six MI hearts (Supplementary Fig. 4). Also, only scattered foci of pH3, but no evidence of condensed chromosomes were observed within these two pH3 positive CM nuclei (Fig. 7c, f), indicating that they were in late G2 phase or early M phase. Thus, we failed to find any solid evidence for progression through M phase or CM proliferation.

### Intronic RNAscope probes *Myl2* and *Myl4* specifically label ventricular CMs and atrial CMs, respectively

CMs generated from ESCs or iPSCs are utilized to model and study heart disease, and are of particularly utility as models for human CMs^48–52^. Identification of CMs subtypes is a critical aspect of this modeling, and having nuclear localized probes would offer the advantage of being able to identify subtypes, and study their biological activities, including proliferation in situ. Thus, we wanted to explore the utility of intronic RNA probes to identify ventricular or atrial CMs, utilizing mouse heart tissue as a proof of principle. Previous genome-wide transcriptional profiling of the four heart chambers and the interventricular septum revealed that myosin light chain 2 (*Myl2*) is strongly and specifically expressed in the ventricles, while myosin light chain 4 (*Myl4*) shows strong and specific expression in the atria^53^. Thus, we designed intronic RNAscope probes for *Myl2* and *Myl4* as specific markers for the ventricles and atria, respectively.

**Fig. 8:**
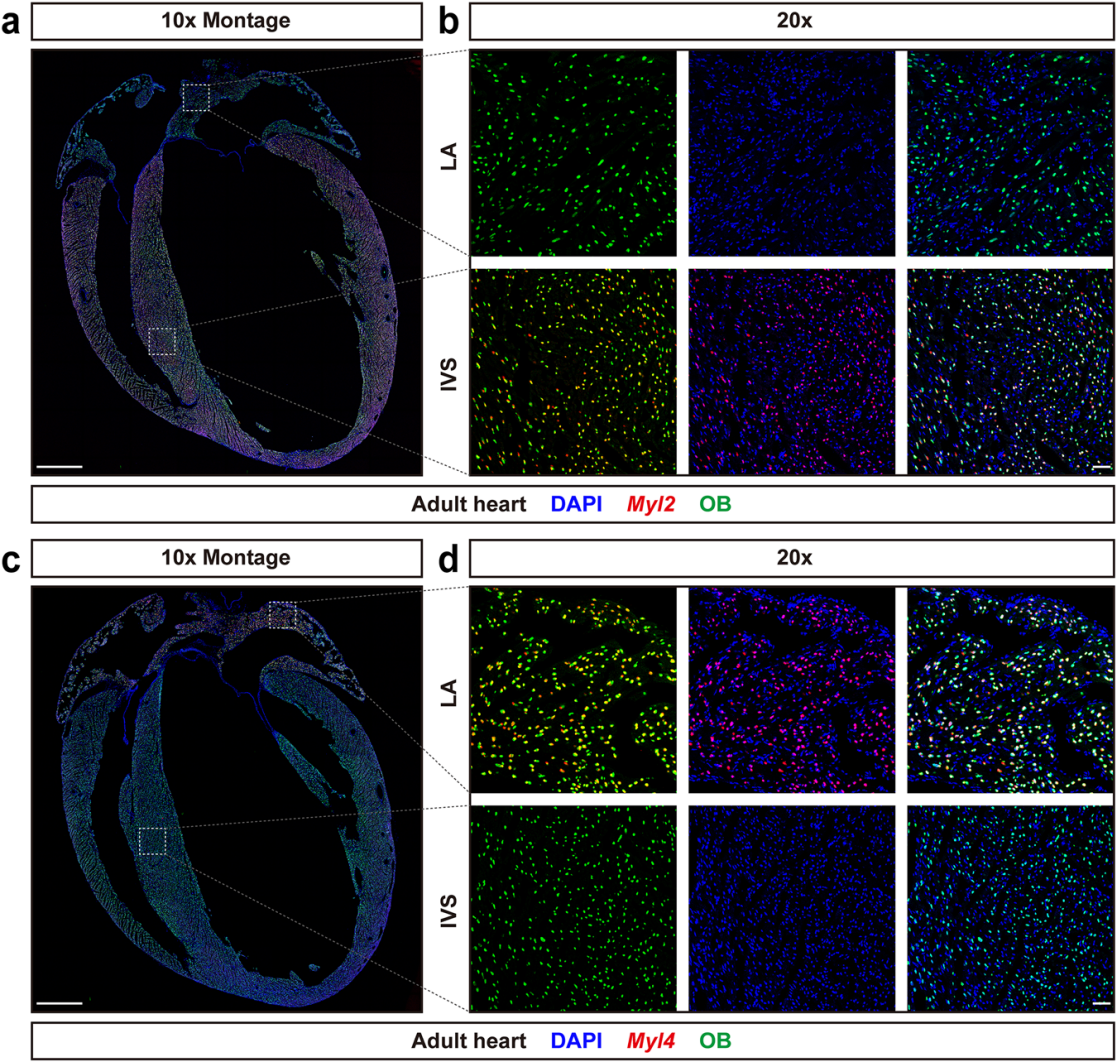
*Myl2* and *Myl4* intronic RNAscope probes label ventricular and atrial CM nuclei, respectively. **a** Obscurin-H2B-GFP fluorescent signals were detected throughout the hearts, while *Myl2* intronic RNAs were only observed in the ventricular region. Scale bar, 1 mm. **b** Representative figures demonstrated *Myl2* intronic RNAs showed a high degree of colocalization with Obscurin-H2B-GFP in ventricular but not atrial region. Scale bar, 100 μm. **c** Obscurin-H2B-GFP fluorescent signals were observed in the whole hearts, while *Myl4* intronic RNAs were only detected in the atrial region. Scale bar, 1 mm. **d** Representative figures demonstrated *Myl4* intronic RNAs showed a high degree of colocalization with Obscurin-H2B-GFP in atrial but not ventricular region. Scale bar, 100 μm. LA left atrium, IVS interventricular septum.

To verify the specificity and efficiency of these probes, we harvested hearts from 8-week-old *Obscn*^GFP/GFP^ male mice and prepared cryosections. RNAscope fluorescence assays with *Myl2*, or *Myl4* intronic RNAscope probes, combined with fluorescence immunostaining for GFP were performed. Strong expression of *Myl2* intronic RNA was observed specifically in ventricular CMs (Fig. 8a). Also, *Myl2* intronic RNAs showed a high degree of colocalization with Obscurin-H2B-GFP (Fig. 8b). We calculated the frequency of ventricular CM nuclei double labeled with *Myl2* intronic RNAscope probe and GFP, using the total number of ventricular CM nuclei labeled with either *Myl2* intronic RNAscope probe or GFP as the reference. These results showed more than 95% of ventricular CM nuclei were double labeled with the *Myl2* intronic RNAscope probe and GFP (Supplementary Fig. 5a). In contrast, *Myl4* intronic RNAs were detected specifically within atrial CMs (Fig. 8c), and displayed a high degree of colocalization with Obscurin-H2B-GFP (Fig. 8d). Quantification indicated that more than 95% of atrial CM nuclei were labeled with both *Myl4* intronic RNAscope probe and GFP (Supplementary Fig. 5b). These results demonstrated that both *Myl2* and *Myl4* intronic RNAscope probes could work efficiently and specifically.

## Discussion

Although CM proliferation in adult hearts at baseline or in response to heart injuries including MI have been reported by several groups, the rate of potential CM proliferation is extremely low^4–6^. In adult hearts, non-CM nuclei account for 70–80% of the total nuclei and can be located in close proximity to CM cytoplasm, which makes it difficult to distinguish non-CM nuclei from CM nuclei^7^. Since interstitial cells are much more likely to be cell cycle active than CMs, unless CM nuclei can be unequivocally identified, this can lead to false positives^7^. Therefore, an ongoing challenge in cardiac regeneration studies is to establish techniques for unequivocal identification of CM nuclei, crucial for accurately identifying CMs undergoing the cell cycle.

Traditional methods for cardiac section analysis have significant limitations. Most groups have used antibodies to sarcomeric proteins (such as α-actinin, myosin heavy chain, cardiac troponins C, I, and T) for CM identification. This method labels only the cytoplasm of CMs but not the nuclei, which can be error-prone. This may partially explain discrepancies in studies concerning the turnover rate of adult mouse CMs and CM regenerative potential after heart injuries^7,8^. Additionally, antibodies to a few nuclear CM markers have been used in analysis of cardiac sections, including Nkx2.5^13–15^, Gata4^7,13^, Mef2c^16^, and PCM1^8,17–19^. However, the application of these markers still has limitations, owing to issues of specificity and signal strength^1,7,13,16,20^. Many of the above markers may fail to be visualized during mitosis upon nuclear envelope breakdown, making it challenging to capture CMs undergoing mitosis. Additionally, post MI, staining for all nuclear antigens in the BZ and IZ is challenging due to high background autofluorescence^8^. However, identification of cell cycle active CMs in these regions is particularly important since the cell cycle activity of CMs in the BZ and IZ is stimulated to a much higher degree compared with the RZ^5,6,22–24^. Genetically modified mouse lines are also used for identification of CM nuclei, including the Obscurin-H2B-GFP^25,26^, αMHC-H2B-mCherry mouse^8,27^, and MHC-nLAC mouse lines^22^. However, genetic manipulations may pose a risk in generating cardiac phenotypes. For example, *Obscn*^GFP/GFP^ mice display alterations in architecture of sarcoplasmic reticulum, and disrupted expression of several sarcoplasmic reticulum associated proteins^25^.

Our *Tnnt2* intronic RNAscope probe overcomes the above shortcomings. *Tnnt2* is the gene that encodes for cardiac troponin T, ensuring the specificity of *Tnnt2* intronic RNAscope probe for CMs. Since intronic RNAs are localized to nuclei, the signal for the *Tnnt2* intronic RNAscope probe is restricted to CM nuclei (Fig. 1a, b). Utlization of pH3 immunostaining and Fucci2a indicator mice demonstrated that the *Tnnt2* intronic RNAscope probe labeled embryonic CM nuclei or chromatin at all stages of mitosis (Figs. 2–4, Supplementary Fig. 2), even when nuclear envelope breakdown occurred, allowing for the identification of CM nuclei during mitosis. At prophase, *Tnnt2* intronic RNAs were strictly colocalized with CM nuclei (Fig. 3a, b). During metaphase and anaphase, some *Tnnt2* intronic RNAs were still colocalized with CM chromatin, while the remainder were distributed peri-chromosomally (Figs. 3e–h, 4, Supplementary Fig. 2). Therefore, *Tnnt2* intronic probes can be used to identify CMs even during late mitotic events.

Compared with immunostaining for CM nuclear antigens, the *Tnnt2* intronic RNAscope probe generated clear signals in BZ and IZ after MI, without high background fluorescence (Figs. 5 and 7). Within all CM nuclei labeled by either *Tnnt2* intronic probe and Obscurin-GFP fluorescent signals, more than 95% of total CM nuclei were double positive for both *Tnnt2* intronic probe and GFP fluorescent signals (Fig. 1), demonstrating the high specificity of the *Tnnt2* intronic RNAscope probe for CM nuclei. Therefore, this probe is likely to be proficient at capturing any cycling CMs after MI. *Tnnt2* intronic RNAscope probe signals are independent of genetic manipulation, allowing for unequivocal identification of CM nuclei without concern for any potential cardiac phenotypes. Additionally, our *Myl2* or *Myl4* intronic RNAscope probes can be used for ventricular or atrial specific CM nuclei identification, respectively (Fig. 8), which may be of particular utility for in vitro generated CMs.

Cell cycle activity or DNA synthesis in CM nuclei after MI has been reported by several groups^4–6^. In many studies, BrdU or EdU administration has been performed to monitor DNA synthesis in BZ and IZ CM nuclei. However, a comprehensive time-course study of DNA synthesis dynamics in CM nuclei post MI has not yet been conducted. BrdU or EdU administration strategies vary significantly among different studies, with duration of administration ranging from 5 days to two weeks, and the starting point from day 1 to as late as 9 weeks post MI^44–47^. We conducted 4-day EdU injections with nine different initial days, including days 2, 4, 6, 8, 10, 12, 14, 16, and 30 post MI surgery, and harvested the hearts two days following the final injection. With the help of the *Tnnt2* intronic RNAscope probe, we observed similar dynamics of DNA synthesis in CM nuclei within both BZ and IZ. Surprisingly, almost no EdU^+^ CM nuclei were detected before day 8 post MI. The highest frequency of EdU^+^ CM was detected between days 12-15 post MI. EdU incorporation in CM nuclei decreased at day 16 post MI, and dropped to nearly 0 by day 30 post MI (Fig. 6, Supplementary Fig. 3). Our studies were performed in FVB/NJ strain background, and these dynamics may be strain background dependent. Nonetheless, our results are likely to provide valuable guidance for other researchers in determining the timing of EdU administration post MI.

We requested assistance from Advanced Cell Diagnostics (ACD) in designing all intronic RNAscope probes. The first *Myl4* intronic probe only generated weak signals, while the second one worked well in labeling atrial CM nuclei. After consulting with ACD, we found the first intronic probe was just 9 ZZ pairs, while the second one was 20 ZZ pairs. In theory, the first intronic probe essentially has 11 fewer opportunities for amplification trees to be formed per target molecule. Furthermore, if there were issues with binding due to RNA quality or suboptimal pretreatment, the signal may have been further diminished. Therefore, ensuring an adequate number of ZZ pairs is essential to guarantee the necessary signal strength. An optimal RNAscope probe should contain 20 ZZ pairs, targeting approximately 1000 bp of target sequence. While shorter probes can also be effective, they offer reduced assurance against redundancy, and other variables may have a more pronounced impact on the signal. When designing intronic probes, it is important that the length of the target sequence is sufficient. While a sequence needs to be long enough to accommodate 20 ZZ pairs (1000 bp), this alone does not guarantee usability. The sequence must also be unique compared to other sequences in the mouse genome and should have an appropriate composition that aligns with the assay conditions. Therefore, simply having a longer sequence does not ensure the feasibility of designing 20 ZZ pairs. Increasing the probe length may enhance signal visualization, but it also carries the risk of increasing the fluorescent background.

Overall, our intronic RNAscope probes have the same CM specificity as sarcomeric proteins but exhibit nuclear localization. They can effectively label CM nuclei or chromatin during mitosis while avoiding potential side effects associated with genetic manipulation. We believe that the application of these intronic RNAscope probes in the field of cardiac science holds great value and promise.

## Supplementary information

Supplementary Information

Description of Additional Supplementary File

Supplementary Data

Reporting summary

## Data Availability

All source data can be obtained from Supplementary Data. Additional data supporting the findings of this study are available from the corresponding author upon reasonable request.
