# Supplementary material for: Intronic RNAscope probes enable precise identification of cardiomyocyte nuclei and cell cycle activity
Source: Commun Biol. 2025 Apr 7;8:577. doi: 10.1038/s42003-025-08012-z (PMC11977257; doi:10.1038/s42003-025-08012-z)
Supplement: Supplementary file 1 — Supplementary Information [file 42003_2025_8012_MOESM1_ESM.docx]

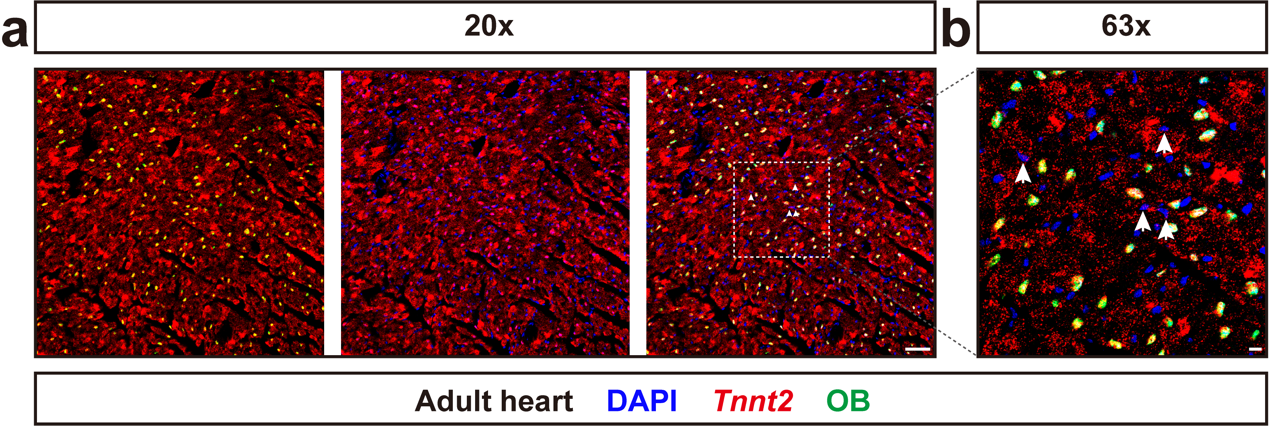


**Supplementary Figure 1 *Tnnt2* exonic RNAscope probe failed to distinguish between CM and interstitial nuclei. a,** *Tnnt2* exonic RNA was detected through the CM cytoplasm, making it difficult to differentiate CM from interstitial nuclei. Scale bar, 100 μm. **b,** using high-magnification imaging, some interstitial nuclei (indicated by arrowheads) partially colocalized with *Tnnt2* exonic RNA. Scale bar, 10 μm.


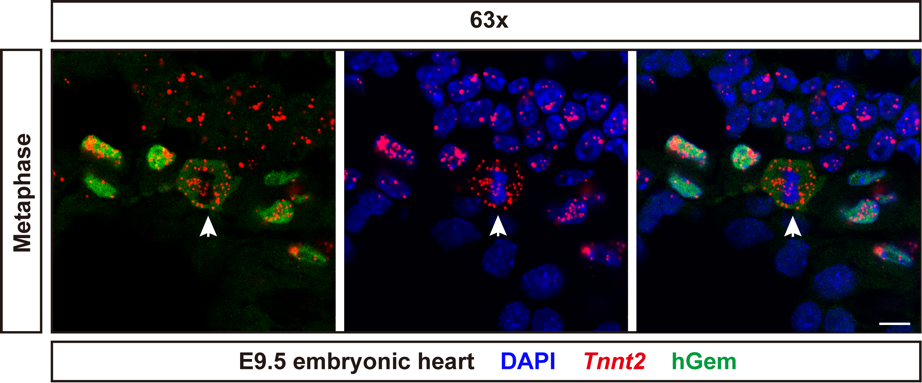
­

**Supplementary** **Figure 2 *Tnnt2* intronic RNAscope probe labels CMs during metaphase.** A metaphase CM was identified by *Tnnt2* intronic RNAscope probe and mVenus-hGem fluorescent signals. Some *Tnnt2* intronic RNAs colocalized with DAPI-labeled CM nuclei, while the remainder were distributed with mVenus-hGem in a peri-chromosomal location. Scale bar, 10 μm.


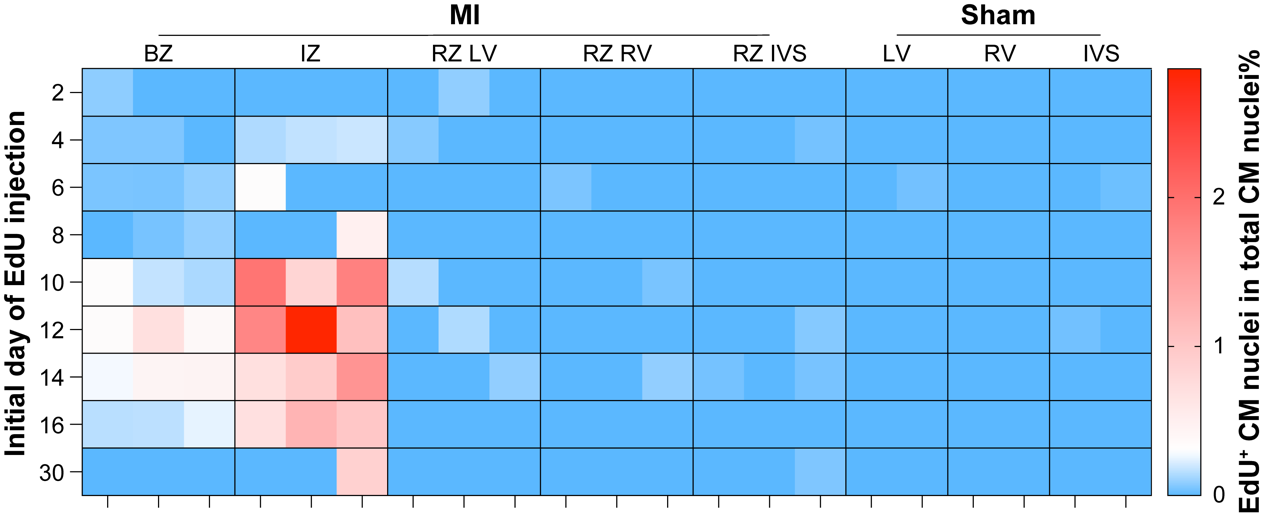


**Supplementary Figure 3 Heat map shows dynamics of EdU incorporation in CM nuclei post MI surgeries in both Sham and MI hearts.** In BZ and IZ, only very rare EdU incorporation in CM nuclei was observed before day 10 post MI. The highest level of EdU incorporation in CM nuclei occurred during days 12-15 post MI. At day 30, EdU incorporation in CM nuclei had nearly ceased. In the RZ and Sham hearts, negligible EdU incorporation was detected in CM nuclei (n=3 for each timepoint for MI; n=2 for each timepoint for Sham). To facilitate comparison with RZ and Sham, the data for BZ and IZ, previously presented in Figures 6D and 6E, are now distinctly represented in this figure. BZ, border zone; IZ, infarct zone; RZ, remote zone; LV, left ventricle; RV, right ventricle; IVS, interventricular septum.


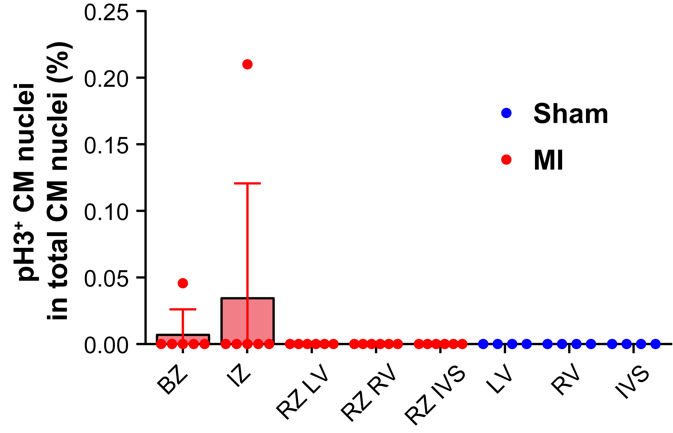


**Supplementary Figure 4 Rare pH3 positive CM nuclei were observed in BZ and IZ post MI surgery.** Only one pH3 positive CM nuclei was observed in both BZ and IZ, respectively. No pH3 positive CM nuclei were detected in either RZ of the MI hearts, or in the Sham hearts (n=4 for Sham; n=6 for MI). Total counts of CM nuclei in MI hearts: 13346 in BZ, 3007 in IZ, 4955 in RZ LV, 11252 in RZ RV, and 9656 in RZ IVS. Total counts of CM nuclei in Sham hearts: 6600 in LV, 8422 in RV, and 7570 in IVS. Negative binomial regression was performed to determine statistical significance. Data are presented as mean ± SD. BZ, border zone; IZ, infarct zone; RZ, remote zone; LV, left ventricle; RV, right ventricle; IVS, interventricular septum.


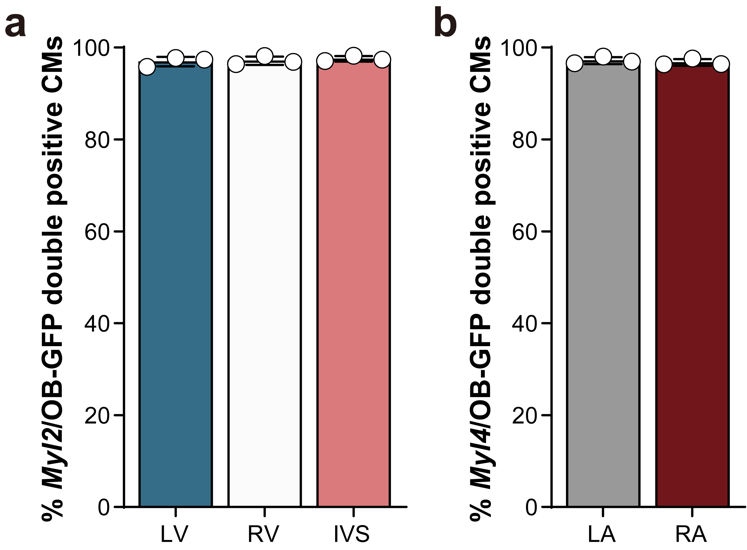


**Supplementary Figure 5 The *Myl2* and *Myl4* intronic RNAscope probes efficiently label ventricular and atrial CM nuclei, respectively. a,** more than 95% of labeled ventricular CM nuclei were positive for both *Myl2* intronic RNAscope probe and Obscurin-H2B-GFP. Three hearts were used for analysis, and in each heart three photos were taken for each region: LV, RV, and IVS. Total counts of CM nuclei: 1135 in LV, 1692 in RV, and 1431 in IVS. **b,** more than 95% of labeled ventricular CM nuclei were positive for both *Myl4* intronic RNAscope probe and Obscurin-H2B-GFP. Three hearts were used for analysis, and in each heart three photos were taken for each region: LA and RA. Total counts of CM nuclei: 1273 in LA and 1133 in RA. Negative binomial regression was performed to determine statistical significance. Data are presented as mean ± SD. LA, left atrium; RA, right atrium; LV, left ventricle; RV, right ventricle; IVS, interventricular septum.

**Supplementary Table 1**

**Number of EdU^+^ CM nuclei and total CM nuclei counted in BZ, IZ and RZ of post MI hearts**

| Initial Day | ID | BZ | | IZ | | RZ LV | | RZ RV | | RZ IVS | |
| --- | --- | --- | --- | --- | --- | --- | --- | --- | --- | --- | --- |
|  |  | EdU^+^ | Total | EdU^+^ | Total | EdU^+^ | Total | EdU^+^ | Total | EdU^+^ | Total |
| Day2 | D2-1 | 2 | 2387 | 0 | 310 | 0 | 336 | 0 | 1596 | 0 | 897 |
|  | D2-2 | 0 | 2300 | 0 | 374 | 1 | 1137 | 0 | 2016 | 0 | 2106 |
|  | D2-3 | 0 | 2966 | 0 | 405 | 0 | 626 | 0 | 2000 | 0 | 2037 |
| Day4 | D4-1 | 1 | 3056 | 1 | 700 | 1 | 1390 | 0 | 2157 | 0 | 2401 |
|  | D4-2 | 0 | 3173 | 1 | 567 | 0 | 1023 | 0 | 1878 | 0 | 2351 |
|  | D4-3 | 1 | 3013 | 1 | 518 | 0 | 1130 | 0 | 2602 | 1 | 2584 |
| Day6 | D6-1 | 1 | 2016 | 2 | 608 | 0 | 443 | 1 | 1955 | 0 | 1489 |
|  | D6-2 | 1 | 2146 | 0 | 416 | 0 | 1049 | 0 | 2363 | 0 | 1857 |
|  | D6-3 | 2 | 2167 | 0 | 461 | 0 | 827 | 0 | 2604 | 0 | 2234 |
| Day8 | D8-1 | 0 | 1884 | 0 | 382 | 0 | 452 | 0 | 1402 | 0 | 1396 |
|  | D8-2 | 1 | 2367 | 0 | 645 | 0 | 482 | 0 | 1817 | 0 | 1932 |
|  | D8-3 | 2 | 2129 | 3 | 624 | 0 | 783 | 0 | 2439 | 0 | 1527 |
| Day10 | D10-1 | 7 | 2124 | 10 | 516 | 1 | 638 | 0 | 1374 | 0 | 1311 |
|  | D10-2 | 4 | 2217 | 4 | 484 | 0 | 1002 | 0 | 2466 | 0 | 1930 |
|  | D10-3 | 3 | 2237 | 13 | 723 | 0 | 826 | 1 | 2171 | 0 | 2181 |
| Day12 | D12-1 | 9 | 2662 | 6 | 345 | 0 | 620 | 0 | 1473 | 0 | 1283 |
|  | D12-2 | 14 | 2044 | 13 | 454 | 1 | 697 | 0 | 2065 | 0 | 2026 |
|  | D12-3 | 8 | 2156 | 8 | 743 | 0 | 292 | 0 | 1458 | 1 | 1488 |
| Day14 | D14-1 | 8 | 2869 | 4 | 586 | 0 | 1265 | 0 | 2765 | 1 | 2575 |
|  | D14-2 | 10 | 2321 | 5 | 534 | 0 | 1081 | 0 | 2142 | 0 | 1897 |
|  | D14-3 | 10 | 2264 | 9 | 568 | 1 | 1108 | 2 | 2249 | 1 | 2278 |
| Day16 | D16-1 | 4 | 2469 | 4 | 588 | 0 | 999 | 0 | 2092 | 0 | 1700 |
|  | D16-2 | 4 | 2399 | 7 | 576 | 0 | 1209 | 0 | 1715 | 0 | 2064 |
|  | D16-3 | 6 | 2391 | 6 | 609 | 0 | 579 | 0 | 2024 | 0 | 1575 |
| Day30 | D30-1 | 0 | 1869 | 0 | 374 | 0 | 761 | 0 | 1710 | 0 | 1313 |
|  | D30-2 | 0 | 1753 | 0 | 303 | 0 | 805 | 0 | 1741 | 0 | 2108 |
|  | D30-3 | 0 | 2161 | 3 | 345 | 0 | 589 | 0 | 1937 | 1 | 1806 |

**Supplementary Table 2**

**Number of EdU^+^ CM nuclei and total CM nuclei counted in Sham hearts**

| Timepoint | ID | LV | | RV | | S | |
| --- | --- | --- | --- | --- | --- | --- | --- |
|  |  | EdU^+^ | Total | EdU^+^ | Total | EdU^+^ | Total |
| Day2 | D2S-1 | 0 | 2573 | 0 | 2178 | 0 | 2803 |
|  | D2S-2 | 0 | 1896 | 0 | 2305 | 0 | 2055 |
| Day4 | D4S-1 | 0 | 2271 | 0 | 2354 | 0 | 2689 |
|  | D4S-2 | 0 | 2054 | 0 | 2829 | 0 | 2317 |
| Day6 | D6S-1 | 0 | 2129 | 0 | 2373 | 0 | 2865 |
|  | D6S-2 | 1 | 2800 | 0 | 2498 | 1 | 3541 |
| Day8 | D8S-1 | 0 | 1986 | 0 | 2366 | 0 | 2818 |
|  | D8S-2 | 0 | 2167 | 0 | 3010 | 0 | 2829 |
| Day10 | D10S-1 | 0 | 2291 | 0 | 2076 | 0 | 2785 |
|  | D10S-2 | 0 | 2010 | 0 | 2804 | 0 | 2839 |
| Day12 | D12S-1 | 0 | 1841 | 0 | 2466 | 1 | 2946 |
|  | D12S-2 | 0 | 1816 | 0 | 2644 | 0 | 2774 |
| Day14 | D14S-1 | 0 | 1885 | 0 | 2698 | 0 | 2572 |
|  | D14S-2 | 0 | 1847 | 0 | 2993 | 0 | 2623 |
| Day16 | D16S-1 | 0 | 2284 | 0 | 2864 | 0 | 3094 |
|  | D16S-2 | 0 | 1953 | 0 | 2471 | 0 | 2801 |
| Day30 | D30S-1 | 0 | 1510 | 0 | 1839 | 0 | 1929 |
|  | D30S-2 | 0 | 1663 | 0 | 2755 | 0 | 2232 |
