# Supplementary material for: Intronic RNAscope probes enable precise identification of cardiomyocyte nuclei and cell cycle activity
Source: Commun Biol. 2025 Apr 7;8:577. doi: 10.1038/s42003-025-08012-z (PMC11977257; doi:10.1038/s42003-025-08012-z)
Supplement: Supplementary file 2 — Description of Additional Supplementary File [file 42003_2025_8012_MOESM2_ESM.pdf]

## Description Of Additional Supplementary File

**File name:** Supplementary Data

**Description:** The source data behind the graphs in the paper
