# Supplementary material for: Intronic RNAscope probes enable precise identification of cardiomyocyte nuclei and cell cycle activity
Source: Commun Biol. 2025 Apr 7;8:577. doi: 10.1038/s42003-025-08012-z (PMC11977257; doi:10.1038/s42003-025-08012-z)
Supplement: Supplementary file 4 — Reporting summary [file 42003_2025_8012_MOESM4_ESM.pdf]

nature portfolio

Corresponding author(s): Sylvia Evans

Last updated by author(s): Mar 13, 2025

Reporting Summary

Nature Portfolio wishes to improve the reproducibility of the work that we publish. This form provides structure for consistency and transparency in reporting. For further information on Nature Portfolio policies, see our Editorial Policies and the Editorial Policy Checklist.

Statistics

For all statistical analyses, confirm that the following items are present in the figure legend, table legend, main text, or Methods section.

n/a	Confirmed	
☐	☒	The exact sample size (*n*) for each experimental group/condition, given as a discrete number and unit of measurement
☐	☒	A statement on whether measurements were taken from distinct samples or whether the same sample was measured repeatedly
☐	☒	The statistical test(s) used AND whether they are one- or two-sided<br>*Only common tests should be described solely by name; describe more complex techniques in the Methods section.*
☒	☐	A description of all covariates tested
☐	☒	A description of any assumptions or corrections, such as tests of normality and adjustment for multiple comparisons
☐	☒	A full description of the statistical parameters including central tendency (e.g. means) or other basic estimates (e.g. regression coefficient) AND variation (e.g. standard deviation) or associated estimates of uncertainty (e.g. confidence intervals)
☐	☒	For null hypothesis testing, the test statistic (e.g. *F*, *t*, *r*) with confidence intervals, effect sizes, degrees of freedom and *P* value noted<br>*Give P values as exact values whenever suitable.*
☒	☐	For Bayesian analysis, information on the choice of priors and Markov chain Monte Carlo settings
☒	☐	For hierarchical and complex designs, identification of the appropriate level for tests and full reporting of outcomes
☐	☒	Estimates of effect sizes (e.g. Cohen's *d*, Pearson's *r*), indicating how they were calculated

Our web collection on statistics for biologists contains articles on many of the points above.

Software and code

Policy information about availability of computer code

Data collection	Leica LAS X
Data analysis	graphpad prism 10.1.2 and SPSS28

For manuscripts utilizing custom algorithms or software that are central to the research but not yet described in published literature, software must be made available to editors and reviewers. We strongly encourage code deposition in a community repository (e.g. GitHub). See the Nature Portfolio guidelines for submitting code & software for further information.

Data

Policy information about availability of data

All manuscripts must include a data availability statement. This statement should provide the following information, where applicable:

- Accession codes, unique identifiers, or web links for publicly available datasets
- A description of any restrictions on data availability
- For clinical datasets or third party data, please ensure that the statement adheres to our policy

All data are included in the manuscript, and no additional data are available.

Research involving human participants, their data, or biological material

Policy information about studies with human participants or human data. See also policy information about sex, gender (identity/presentation), and sexual orientation and race, ethnicity and racism.

Reporting on sex and gender	n/a
Reporting on race, ethnicity, or other socially relevant groupings	n/a
Population characteristics	n/a
Recruitment	n/a
Ethics oversight	n/a

Note that full information on the approval of the study protocol must also be provided in the manuscript.

Field-specific reporting

Please select the one below that is the best fit for your research. If you are not sure, read the appropriate sections before making your selection.

☒ Life sciences ☐ Behavioural & social sciences ☐ Ecological, evolutionary & environmental sciences

For a reference copy of the document with all sections, see nature.com/documents/nr-reporting-summary-flat.pdf

Life sciences study design

All studies must disclose on these points even when the disclosure is negative.

Sample size	In Figures 6/S3, 2 Sham and 3 MI animals were used per timepoint. In Figures 7/S4, 4 Sham and 6 MI animals were used. All other figures analyzed 3 animals. The sample size was determined based on ensuring representation from at least three litters and analyzing at least three animals per group to account for biological variability. However, for Figures 6 and S3, only two animals from two litters were used for Sham groups due to the high number of timepoints and prior literature showing minimal EdU-positive cardiomyocytes in Sham hearts. This approach balanced feasibility and data quality while aligning with previous findings.
Data exclusions	Animals were included only if myocardial infarction (MI) was successfully induced during surgery, as evidenced by infarction. Animals without infarction were excluded from the study. This criterion was established a priori. In Figures 6 and S3, 3 mice in the MI groups were excluded due to almost no infarction being generated, indicating surgery failure.
Replication	Each type of experiment was performed at least three times, and the results were consistent.
Randomization	For each litter, animals were randomly assigned to either the Sham or MI group. The order of MI and Sham surgeries was randomized, and all animals were housed in the same room and on the same rack to minimize environmental confounders.
Blinding	During the allocation, the researchers performing the surgeries were unaware of the assigned timepoints for the animals. In addition, the images were analyzed with the timepoints blinded by randomizing the order of the samples. Outcome assessors were not aware of the experimental groups until the data were analyzed and results were generated.

Reporting for specific materials, systems and methods

We require information from authors about some types of materials, experimental systems and methods used in many studies. Here, indicate whether each material, system or method listed is relevant to your study. If you are not sure if a list item applies to your research, read the appropriate section before selecting a response.

Materials & experimental systems

n/a	Involved in the study
☐	☒ Antibodies
☒	☐ Eukaryotic cell lines
☒	☐ Palaeontology and archaeology
☐	☒ Animals and other organisms
☒	☐ Clinical data
☒	☐ Dual use research of concern
☒	☐ Plants

Methods

n/a	Involved in the study
☒	☐ ChIP-seq
☒	☐ Flow cytometry
☒	☐ MRI-based neuroimaging

Antibodies

Antibodies used	Anti-GFP antibody (Abcam, ab13970); Anti-Phospho-Histone H3 (Ser10) antibody (MilliporeSigma, 06-570); Donkey anti-Rabbit IgG (H+L) Highly Cross-Adsorbed Secondary Antibody, Alexa Fluor™ Plus 488 (Thermo Fisher Scientific, A32790)
Validation	These antibodies worked well for mouse tissues based on our data and the validation statement on the manufacturer's website.

Animals and other research organisms

Policy information about studies involving animals; ARRIVE guidelines recommended for reporting animal research, and Sex and Gender in Research

Laboratory animals	We used the FVB/NJ (#001800, The Jackson Laboratory), Obscurin-H2B-GFP, Fucci2a, and XMLC2-Cre mouse lines for our study. All mice used in this study were backcrossed with FVB/NJ mice for a minimum of six generations.
Wild animals	n/a
Reporting on sex	Female mice were used for cardiomyocyte isolation (Figure 1D), while male mice were used for the other experiments.
Field-collected samples	n/a
Ethics oversight	Animal studies were conducted in strict compliance with protocol (S04150) approved by the Institutional Animal Care and Use Committee of the University of California, San Diego (UCSD) and the Guide for the Care and Use of Laboratory Animals published by the National Institutes of Health.

Note that full information on the approval of the study protocol must also be provided in the manuscript.

Plants

Seed stocks	n/a
Novel plant genotypes	n/a
Authentication	n/a
